# Previous Lung Diseases and Lung Cancer Risk: A Systematic Review and Meta-Analysis

**DOI:** 10.1371/journal.pone.0017479

**Published:** 2011-03-31

**Authors:** Darren R. Brenner, John R. McLaughlin, Rayjean J. Hung

**Affiliations:** 1 Samuel Lunenfeld Research Institute of Mount Sinai Hospital, Toronto, Canada; 2 The Dalla Lana School of Public Health, University of Toronto, Toronto, Canada; 3 Cancer Care Ontario, Toronto, Canada; University of Cape Town, South Africa

## Abstract

**Background:**

In order to review the epidemiologic evidence concerning previous lung diseases as risk factors for lung cancer, a meta-analysis and systematic review was conducted.

**Methods:**

Relevant studies were identified through MEDLINE searches. Using random effects models, summary effects of specific previous conditions were evaluated separately and combined. Stratified analyses were conducted based on smoking status, gender, control sources and continent.

**Results:**

A previous history of COPD, chronic bronchitis or emphysema conferred relative risks (RR) of 2.22 (95% confidence interval (CI): 1.66, 2.97) (from 16 studies), 1.52 (95% CI: 1.25, 1.84) (from 23 studies) and 2.04 (95% CI: 1.72, 2.41) (from 20 studies), respectively, and for all these diseases combined 1.80 (95% CI: 1.60, 2.11) (from 39 studies). The RR of lung cancer for subjects with a previous history of pneumonia was 1.43 (95% CI: 1.22–1.68) (from 22 studies) and for subjects with a previous history of tuberculosis was 1.76 (95% CI = 1.49, 2.08), (from 30 studies). Effects were attenuated when restricting analysis to never smokers only for COPD/emphysema/chronic bronchitis (RR = 1.22, 0.97–1.53), however remained significant for pneumonia 1.36 (95% CI: 1.10, 1.69) (from 8 studies) and tuberculosis 1.90 (95% CI: 1.45, 2.50) (from 11 studies).

**Conclusions:**

Previous lung diseases are associated with an increased risk of lung cancer with the evidence among never smokers supporting a direct relationship between previous lung diseases and lung cancer.

## Introduction

Lung Cancer is the most common cancer and the overall leading cause of cancer-related mortality worldwide leading to greater than a million deaths annually [1]. Recent evidence suggests that inflammatory processes may play a central role in carcinogenesis [2],[3],[4],[5]. Previous lung diseases/conditions such as chronic obstructive pulmonary disease (COPD) (emphysema and chronic bronchitis), pneumonia and tuberculosis are major sources of inflammation in lung tissue [6], [7]. These conditions may act as intermediates or catalysts in the development of lung neoplasms and appear to be related to lung cancer development through common etiologies and/or exposures [8]. The combined prevalence of previous lung conditions is high across populations and as such they may be important sources of increased lung cancer risk [9],[10], particularly among never smokers.

The associations between COPD, (emphysema and/or chronic bronchitis), pneumonia and tuberculosis and lung cancer have been investigated previously, however, the evidence is inconclusive due to inconsistent findings and small sample sizes - 65% of the studies identified having less than 500 cases. We therefore conducted a systematic review of the scientific literature in order to conduct a meta-analysis of the associations between COPD, emphysema, chronic bronchitis, pneumonia, tuberculosis and lung cancer risk. The main issue in investigating previous lung diseases and lung cancer risk is the possible confounding by smoking. In this analysis, we focused on the direct effects of disease by addressing the potential role of confounding from smoking in these associations with lung cancer. This was assessed through study eligibility and subgroup analysis in studies of never smokers.

### Previous Lung Diseases

COPD is characterized by airflow obstruction in the lungs and the related symptoms that impede the normal expiratory volume of the lungs [11]. COPD most commonly refers to patients with emphysema (the enlargement and destruction of the alveoli) and/or chronic bronchitis (chronic inflammation and scarring of bronchi) [12]. The condition has been defined in several ways, and the differences in definitions and diagnosis affect the estimates of the burden of the disease. The most common definitions involve either airflow limitation (American Thoracic Society) or reduced maximum expiratory flow (European Respiratory Society) which is progressive and mostly irreversible. The Global Initiative for Chronic Obstructive Lung Disease (GOLD) defines the disease in stages of clinical severity based on forced expiratory volume (FEV_1_ & FEV_1_/FVC) from post-bronchodilator spirometry [13]. The disease affects a large proportion of adults with prevalence estimates varying from 4.3% to 5.9% in the US adult population [14]. COPD has been associated with active tobacco smoking (attributable risk estimates in the range of 45% (UK) and 50% (US) among adults) [15], [16], however, chronic bronchitis and less frequently emphysema are also observed among lifetime nonsmokers (chronic bronchitis prevalence among nonsmokers varies across populations 6.3–12.1% [17], [18]). The incidence rate of COPD among never smokers increases with age to approximately 10–12% by age 75 in males and to approximately 20% in by age 75 in females [19].

Pneumonia is an infection of the lungs and respiratory tract most often caused by viruses, bacteria and other organisms. Infection is quite common among adults and pneumonia incidence is highest in the elderly and very young where immune systems are compromised. The highest hospital discharge rate for pneumonia per age group in the US was among those 65 and over at 221.3 per 10,000 [20]. The most common method of clinical diagnosis for pneumonia employs the use of serum antibody determination, particularly microimmunofluorescence [21].

Tuberculosis, another type of infection affecting the lungs is caused by mycobacteria, predominantly Mycobacterium tuberculosis. The incidence of tuberculosis among industrialized countries is approximately 23 cases per 100,000, much lower than the 100–230 cases per 100,000 in other developing countries [22], [23]. Although mortality due to tuberculosis is low in industrialized countries, inflammation and ensuing lung remodeling has been hypothesized to lead to lung cancer development [24], [25], [26].

## Materials and Methods

### Literature Review

We conducted a literature search using the MEDLINE database (US National Library of Medicine) from January 1960 to August 2010 to obtain a comprehensive list of publications containing risk estimates describing the association between lung cancer and previous lung diseases including COPD, emphysema, chronic bronchitis, pneumonia and tuberculosis. Two independent reviewers conducted literatures searches and data abstraction. We utilized the Medical Subject Headings “COPD” or “chronic obstructive pulmonary disease” or “emphysema” or “chronic bronchitis” or “pneumonia” or “tuberculosis” or “respiratory tract diseases” or “lung diseases” and “lung neoplasm” or the text word terms “previous lung disease” and “lung cancer”. Titles and abstract were reviewed for article relevance. In the detailed review of relevant papers effect estimates were extracted including odds ratios, relative risks (RR), hazard ratios (HR) and their corresponding 95% confidence intervals from all included studies. When the same population was examined in multiple publications, we included only the estimate with the largest number of cases reported. Where studies reported estimates for both the total population and among only never smokers within that population [17], [28], [29], [30], the total population estimates were used to combine estimates in all cases except subgroup analysis among never smokers.

Studies were excluded if (i) estimates were not adjusted for smoking status [31], [32], [33], [34], [35], [36], [37], [38] given the strong potential for confounding by smoking; (ii) effect estimates for individual conditions were not reported in the paper [39], [40]; (iii) estimates were based on symptoms only rather than the actual diagnoses [41], [42]; (iv) no diagnostic cut point was provided that could be used to combine the studies (e.g., only estimates for percentiles of lung function scores compared with the reference group of highest lung function were provided) [43], [44].

Given that COPD is a term generally used to describe emphysema and chronic bronchitis, the inflammation and enlargement of air sacs in the lungs, resulting in reduced or limited airflow [12], the meta-analysis was based on estimates reported for these three conditions/classifications combined as well as reported separately (i.e., COPD, emphysema, chronic bronchitis).

Data collection and diagnostic criteria varied across studies and conditions. For COPD, most of the studies collected data based on self-reported condition from questionnaires (i.e. “Did a doctor ever diagnosis you with…?”), while four several used 1-second forced expiratory volume over forced vital capacity (FEV_1_/FVC) or the percent of the predicted forced expiratory volume in 1 second (% FEV_1_) [45], [46], [47], [48], [49]. Emphysema was defined in most studies by self-reported condition, however, a small number of studies employed either quantitative CT scan [45], [50] or radiographic evidence [51]. Pneumonia was defined by self-reported condition or by microimmunofluorescence [52], [53], [54], [55] examining for the levels of IgA antibodies for the *C. pneumoniae* bacteria. Tuberculosis was assessed by self-reported history or X-ray [56].

### Statistical Methods

Random effects models were employed for all meta-analyses [57]. For all previous lung diseases and all subgroups, the potential for publication bias was evaluated by funnel plots and the methods described by Egger [58] et al and Begg et al [59]. Heterogeneity was evaluated using Cochrane's Q-statistic test [60] and the *I* ^2^ statistic [61]. Where there was evidence of heterogeneity across studies, the source of heterogeneity was evaluated by meta-regression (Continent, smoking status, diagnostic method, gender, date study completed, study design and control type used as predictors) and by stratified analysis on smoking status, type of controls, method of diagnosis, study period and gender. If the heterogeneity could not be accounted for by the different characteristics, an influence analysis was conducted to evaluate the source of heterogeneity from single studies by a Galbraith plot and evaluating changes in Q statistics upon study removal. Analyses were also conducted based on “never smokers only” to eliminate possible confounding by smoking. Studies are classified as never smokers where the population consisted exclusively of never smokers by design. A latency analysis was conducted based on study eligibility where studies that excluded persons with a lung disease diagnosis >2, >10 and >20 years before diagnosis were examined across groups. Analyses were conducted using Comprehensive Meta-Analysis Software Version 2 (CMA, NJ), and STATA software version 10 (STATA, College Station TX).

## Results

In total 39 studies were identified that examined the effects of COPD, chronic bronchitis and/or emphysema with estimates adjusted for smoking (Table S1). Specifically, there were 16 studies with estimates of COPD on lung cancer risk; 20 with emphysema and 23 with chronic bronchitis (59 total estimates based on 39 studies). Among the 39 studies, there were 18 population-based and 12 hospital-based case-control studies, 1 mixed case-control study and 8 cohort studies. Out of the 39 studies, 13 studies presented estimates among never smokers only, which reported 23 estimates for various conditions. For pneumonia we identified 22 studies in total, including 10 studies with never smokers only. For tuberculosis, we identified a total of 30 studies including 12 studies with populations of never smokers.

### COPD/emphysema/chronic bronchitis

**Figure 1:**
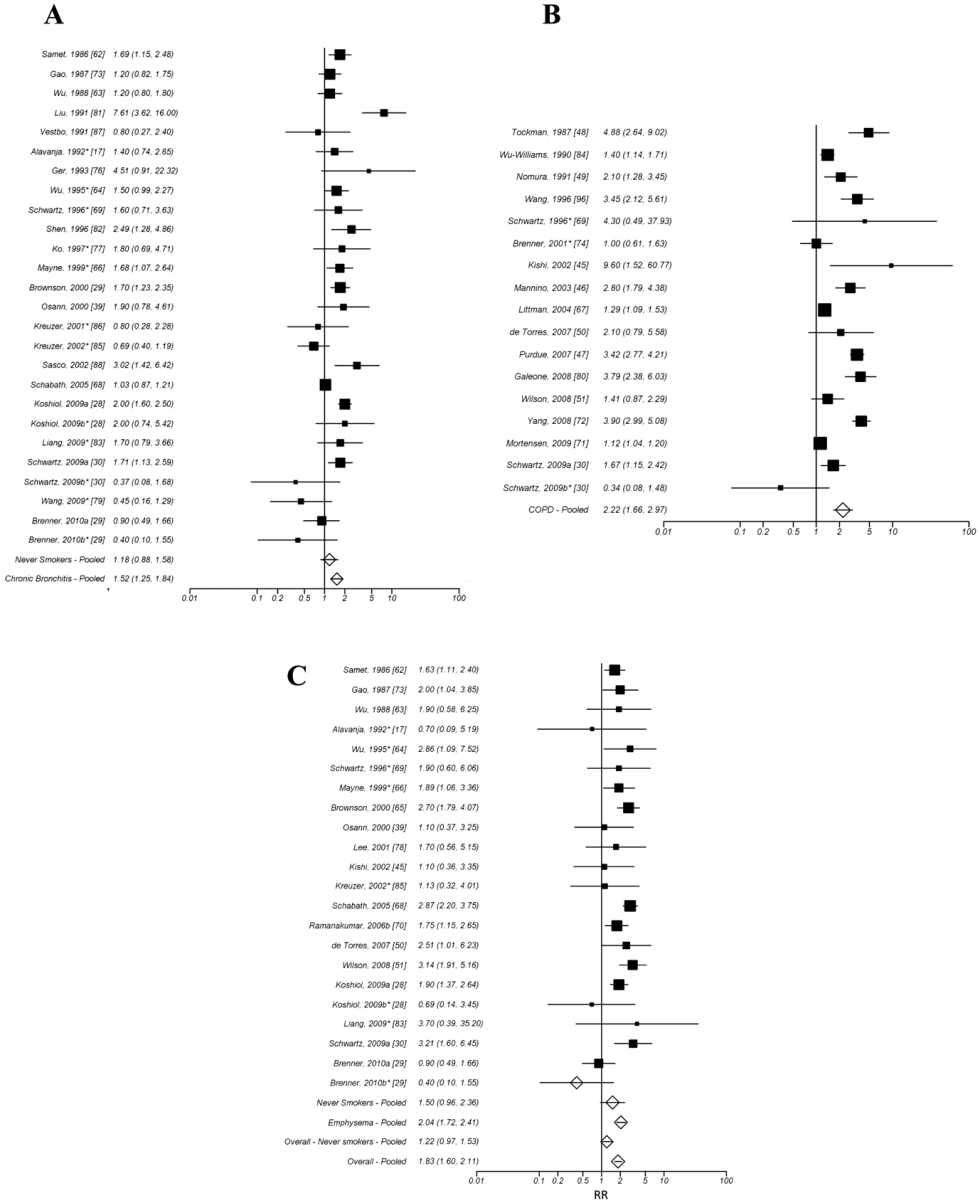
Pooled estimates of the risk associated with a previous diagnosis of COPD, separated by condition and overall with 95% confidence intervals. **A** - study-specific and pooled estimates for chronic bronchitis. **B** - study-specific and pooled estimates for COPD. **C** study-specific and pooled estimates for Emphysema. The estimate labeled Overall – Pooled in panel C represents the combined effects across all three disease groups. *RR* relative risk. The pooled RRs were estimated from random effects models. *Studies of never smokers. The study labeled Ramanakumar, 2006b [70] represents the estimates for one population in study combined among males and females (no combined estimate originally provided). The studies noted with a b* represent the estimates from a subgroup of never smokers presented in the manuscript which were not included in the overall estimates.

Thirty-nine studies examined the relationship between COPD and/or chronic bronchitis and/or emphysema and lung cancer while adjusting for smoking (Table S1). Nineteen of the studies were conducted in North America [17], [29], [30], [39], [45], [46], [48], [51], [62], [63], [64], [65], [66], [67], [68], [69], [70], [71], [72], 12 were conducted in Asia [73], [74], [75], [76], [77], [78], [79], [80], [81], [82], [83], [84], 6 in Europe [28], [47], [50], [85], [86], [87] and 1 in Africa [88]. The combined relative risk (RR) of lung cancer based on all 59 effect estimates was 1.83 (95% confidence interval (CI): 1.60, 2.11). Figure 1 displays a forest plot of the association with estimates separated by each condition (chronic bronchitis, emphysema, COPD as reported in the publication) as well as all conditions combined. It is noteworthy that in examining only those studies that employed a physiological diagnosis of COPD from FEV testing or radiographic evidence of emphysema, the RR was elevated compared to self-reported diagnoses (RR = 2.64, 95% CI: 2.01, 3.47). Among never smokers we did not observe a significant association of all COPD, emphysema and chronic bronchitis estimates combined (RR = 1.22, 95% CI: 0.97, 1.53), however, when the outlying study was removed from the analysis, the effect was 1.29, 95% CI: 1.02–1.63.

In terms of disease-specific estimates, the overall RRs for of COPD, emphysema and chronic bronchitis were 2.22 (95% CI: 1.66, 2.97), 2.04 (95% CI: 1.72, 2.41) and 1.52 (95% CI: 1.25, 1.84), respectively. When restricted to never smokers, associations were not significant for emphysema based on 8 studies (RR = 1.50, 95% CI: 0.96, 2.36), or for chronic bronchitis based on 12 studies (RR = 1.18, 95% CI: 0.88, 1.58). Since passive smoking may confound the association between COPD and lung cancer, sensitivity analyses focused on the four studies that adjusted for SHS exposure [17], [64], [66], [74]. Pooling estimates for COPD, chronic bronchitis/emphysema, chronic bronchitis or emphysema among never smokers adjusted for passive smoking, the RR was 1.49, 95% CI: 1.20, 1.85) (data not shown). Significant heterogeneity was observed among estimates across studies all 59 estimates as well as among several subgroups including when stratified by control type, there was also significant heterogeneity among different control types and when stratified by smoking status (Table 1). When comparing across pooled estimates, significant heterogeneity was observed between control types (p = 0.009) and between continents (p<.001). Meta-regression suggested that study design, control type, smoking status and diagnostic method were predictors of effect size and contributed to heterogeneity (p<0.05). Continent, gender and time of study did not contribute to heterogeneity (Results not shown, disease specific Galbraith plots included in Figures S1, S2, S3, S4). Tests for publication bias among all estimates combined were suggestive of an absence of smaller studies for COPD (Figures S7, S8, S9, S10). To summarize, the pooled estimates among studies examining COPD, emphysema and chronic bronchitis suggest that these factors are associated with a significantly increased risk of lung cancer. Among studies examining never smokers, significant effects were not observed, however, were when one outlying study was removed and among those studies of never smokers that adjusted for SHS exposure. Heterogeneity was observed overall, however, differences across studies can be at least partially explained by study design, control type, smoking status and diagnostic method.

**Table 1:** Results of the meta-analyses of previous lung diseases overall, by condition, study design/control type and among never smoking studies.

Previous Lung Disease		No. of estimates	RR^a^	95% CI	Heterogeneity p-value, I^2^	Comparison across groups p-value	Begg test p-value	Egger test p-value
COPD, or CB/E, or	Overall	59	1.83	1.60, 2.11	<.0001, 84.14		0.58	0.002
Emphysema or	Cohort	10	1.91	1.34, 2.72	<.0001, 93.07	0.88	0.80	0.84
Chronic Bronchitis	Case-control	49	1.82	1.56, 2.11	<.0001, 76.00		0.68	0.32
	Population based	32	1.80	1.57, 2.04	<.0001, 54.26	0.009	0.24	0.88
	Hospital based	15	2.02	1.32, 3.09	<.0001, 88.48		0.32	0.25
	Never smokers	23	1.22	0.97, 1.53	0.05, 34.88		0.41	0.27
	Quantitative diagnosis	10	2.64	2.01, 3.47	0.02, 56.03		0.72	0.41
	North America	35	1.80	1.53, 2,12	<.0001, 83.83	<.0001	0.73	0.001
	Europe	9	1.63	1.11, 2.40	0.012, 81.78		0.55	0.07
	Asia	14	2.01	1.43, 2.81	<.0001, 76.80		0.66	0.26
COPD	Overall	16	2.22	1.66, 2.97	<.0001, 93.55		0.62	0.008
	Cohort studies	7	1.86	1.25, 2.77	<.0001, 94.79		0.55	0.15
	Physiological diagnosis	7	2.73	1.94, 3.83	0.008, 65.36		0.88	0.73
Chronic Bronchitis	Overall	23	1.52	1.25, 1.84	<.0001, 69.92		0.79	0.31
	Never smokers	12	1.18	0.88, 1.58	0.06, 42.04		0.11	0.13
								
Emphysema	Overall	20	2.04	1.72, 2.41	0.12, 28.52		0.50	0.15
	Never smokers	8	1.50	0.96, 2.36	0.30, 6.06		0.27	0.29
Pneumonia	Overall	22	1.43	1.22, 1.68	<.0001, 77.38		0.80	0.51
	Population controls	13	1.53	1.22, 1.92	<.0001, 78.32	0.008	0.63	0.33
	Hospital controls	7	1.46	1.12, 1.90	0.006, 67.10		0.55	0.62
	Never smokers	8	1.36	1.10, 1.69	0.13, 34.84		0.86	0.42
	Serological diagnosis	3	1.74	1.27, 2.38	0.49, 0.00		0.30	0.31
	North America	12	1.50	1.22, 1.70	<.0001, 76.78	0.10	0.50	0.66
	Europe	8	1.18	0.89, 1.56	<.0001, 75.39		1.00	0.32
	Asia	4	1.84	1.37, 2.46	0.96, 0.00		1.00	0.87
Tuberculosis	Overall	30	1.72	1.46, 2.05	0.001, 51.21		0.01	0.002
	Population controls	20	1.53	1.29, 1.81	0.06, 36.00	0.02	0.02	0.02
	Hospital controls	8	2.50	1.69, 3.71	0.05, 50.84		0.90	0.53
	Never smokers	11	1.90	1.45, 2.50	0.29, 14.48		0.11	0.03
	North America	11	1.59	1.17, 2.16	0.14, 32.57	0.37	0.35	0.007
	Europe	4	1.44	0.93, 2.23	0.34, 11.57		1.00	0.61
	Asia	15	1.96	1.54, 2.50	<0.001, 63.30		0.01	0.02
	Asian never smoking women	5	2.23	1.38, 3.61	0.16, 39.80		0.22	0.03

*CB/E* chronic bronchitis and/or emphysema, *CI* confidence interval, *COPD* chronic obstructive pulmonary disease, *RR* relative risk. Where heterogeneity was observed within groups, p values for heterogeneity across groups were calculated.

a The pooled RR were estimated from random effects models.

### Pneumonia

Twenty-two studies examined the relationship between pneumonia and lung cancer risk while adjusting for smoking (Table S1). Eleven studies were conducted in North America [17], [53], [63], [64], [65], [67], [68], [69], [70], [84], [89], 7 in Europe [27], [52], [54], [85], [86], [90], [91], and 4 in East Asia [73], [74], [79], [84]. A significant increase in lung cancer risk was observed among all studies (overall RR = 1.43, 95% CI: 1.22, 1.68). The effect was similar for all studies combined compared to studies with never smokers only (RR = 1.36, 95% CI: 1.10, 1.69).

**Figure 2:**
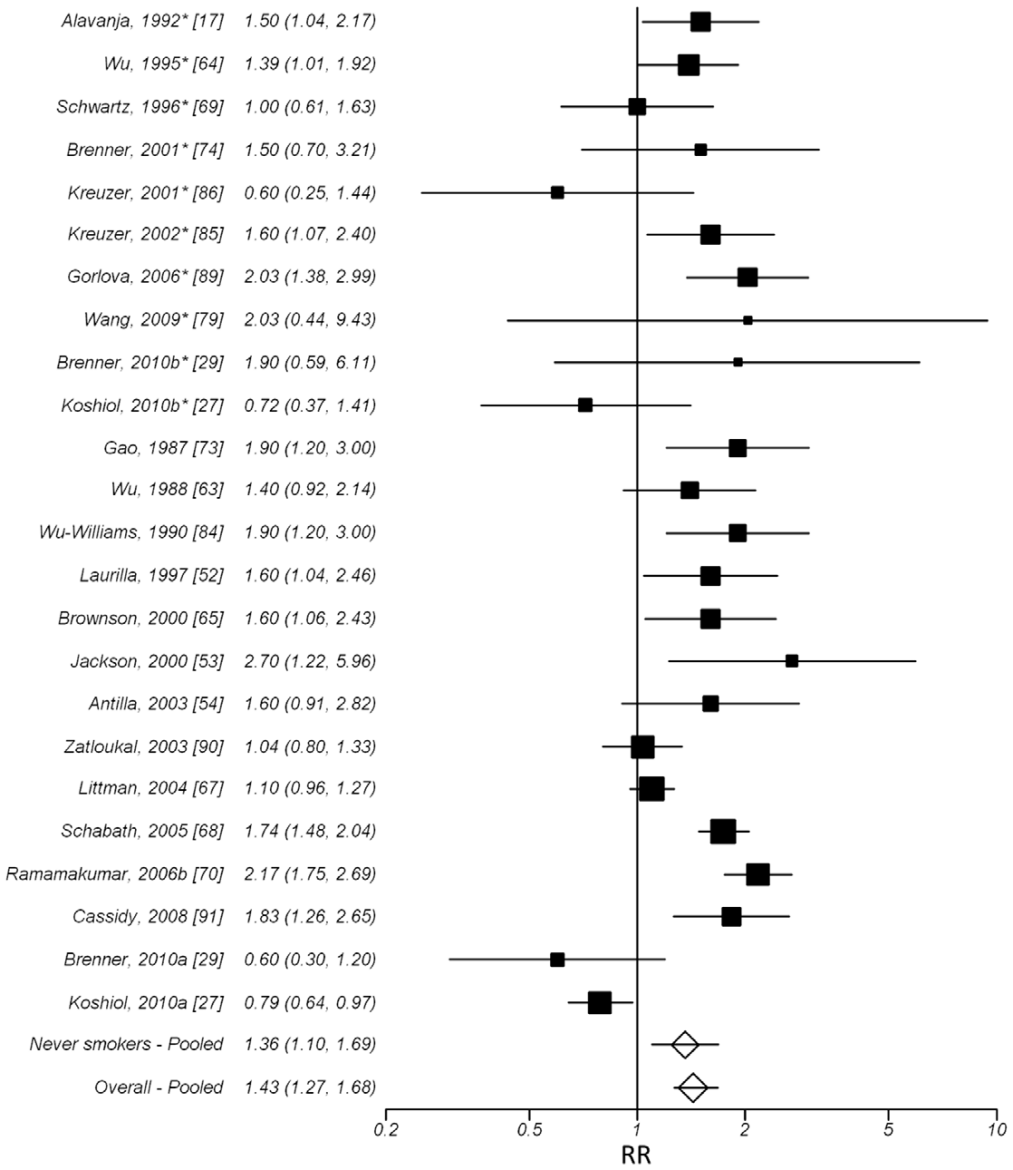
Pooled estimates of the risk associated with a previous diagnosis of pneumonia, separated by smoking status (never smokers on top, smokers on bottom) and overall. *Studies of never smokers. The pooled RRs were estimates from random effects models. The study labeled Ramanakumar, 2006b [70] represents the estimates for one population in study combined among males and females (no combined estimate originally provided). The studies noted with a b* represent the estimates from a subgroup of never smokers presented in the manuscript which were not included in the overall estimates.

The combined effects across all studies separated by participant smoking status are displayed in forest plot format in Figure 2. There was no evidence suggestive of publication bias for this association (Table 1, Funnel plot included in Figure S11). There was significant heterogeneity across all studies (p<.001, Galbraith plot included in Figure S5). However, the heterogeneity diminished when restricting the analysis to never smokers (p = 0.13). Meta-regression suggested that study design, and continent were predictors of effect size and contributed to heterogeneity (p<0.05). In summary, a previous diagnosis of pneumonia across studies was associated with increased lung cancer risk independent of smoking status, with no evidence of publication bias.

### Tuberculosis

**Figure 3:**
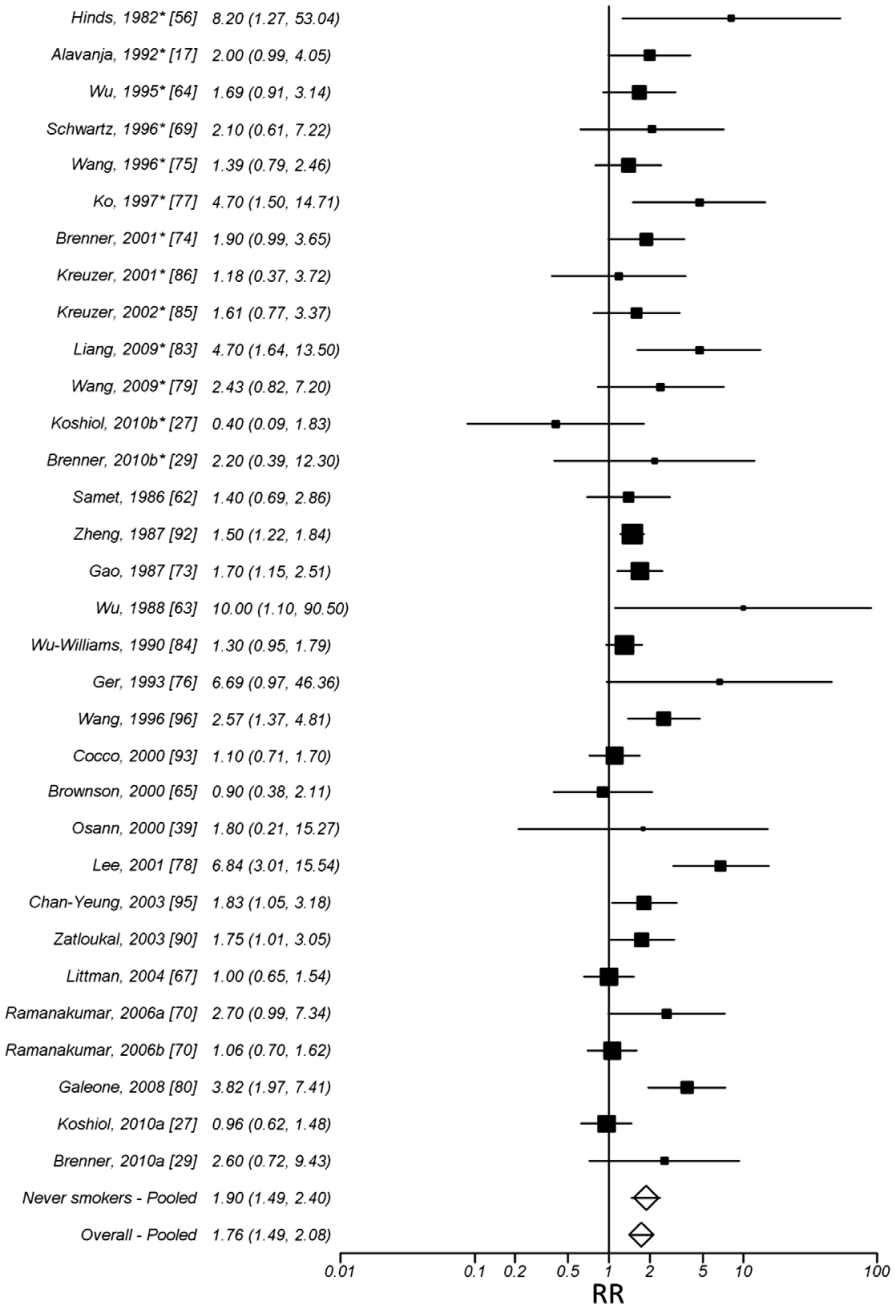
Pooled estimates of the risk associated with a previous diagnosis of tuberculosis, separated by smoking status (never smokers on top, smokers on bottom) and overall. * Studies of never smokers.The pooled RR were estimated from random effects models. The study by Ger 1993 [76] represents only the estimate using population controls was included. The study labeled Ramanakumar, 2006a [70] represents the estimates for one population in study using population controls (cancer controls not included), the studies labeled Ramanakumar, 2006b [70] represents the estimates for the second population in the manuscript study [70] combined among males and females (no combined estimate originally provided). The study by Chan-Yeung, 2003 [90] represents the estimate combined among males and females (no combined estimate originally provided). The studies noted with a b* represent the estimates from a subgroup of never smokers presented in the manuscript which were not included in the overall estimates.

Thirty studies examined the relationship between tuberculosis and lung cancer while adjusting for smoking (Table S1). Eleven of the studies were conducted in the North America [17], [29], [39], [56], [62], [63], [64], [65], [67], [69], [70], 15 were conducted in Asia [73], [74], [75], [76], [77], [78], [79], [80], [83], [84], [92], [93], [94], [95], [96] and 4 were conducted in Europe [27], [85], [86], [90]. The observed effect across all the identified studies suggests an increased risk of lung cancer from tuberculosis (RR = 1.76, 95% CI: 1.49, 2.08). The effect was similar for all studies when compared to only never smokers with the effect of tuberculosis among never smokers being slightly elevated (RR = 1.90, 95% CI: 1.45, 2.50). The combined effects across all studies as well as separated by participant smoking status are displayed in forest plot format in Figure 3. The gender specific results showed very similar effects for men and women. Heterogeneity was observed across all tuberculosis studies combined (p<.001, Galbraith plot included in Figure S6) as well as among studies that examined populations of smokers (p<.001). Among never smokers, no heterogeneity was observed (p = 0.29). Publication bias was observed across all studies of tuberculosis (Funnel plot included in Figure S12), however, not among studies examining never smokers. In summary, a previous diagnosis of tuberculosis was associated with increased lung cancer risk across studies independent of smoking. Heterogeneity was observed across all studies, however, can be partially attributed to differences in controls, continent and smoking status and gender of participants (Table 1).

## Discussion

In this meta-analysis, an increased risk of lung cancer was observed for COPD, emphysema, chronic bronchitis, pneumonia and tuberculosis when examining the studies that adjusted analyses for smoking. Of particular interest are the significant effects also observed among never smokers for pneumonia and tuberculosis. For chronic bronchitis and emphysema, the combined estimates were lower among never smokers, indicating that residual confounding from tobacco may explain some of the effect among smokers, however, does not appear to fully explain the association.

We estimated the combined effects of previous lung diseases on lung cancer risk in studies where effects were adjusted for smoking. Although, the precision of adjustment varied from study to study as seen in the detailed adjustment column of the study table, in essence the estimates provided in this meta-analysis represent the direct effects of the diseases adjusted for smoking status. It is possible that residual confounding of smoking could account for part of the association observed. Nevertheless, the effects among never smokers suggest these previous lung diseases such as pneumonia and tuberculosis have independent effect on lung cancer risk.

It is difficult to conclude from our results whether it is indeed the inflammatory sequelae of these diseases that increase lung cancer risk or whether it is the pathogenesis of the diseases themselves. While it is likely that the diseases of interest in this investigation act in different biological causal pathways, if acting independent of tobacco exposure, however, the inflammatory response is the most likely common causal link [97].

There are several sources of bias that must be addressed in the conduct and design of the individual studies as well as in the synthesis of the studies. The majority of studies reported were case-control studies in which data concerning previous lung diseases were abstracted or collected via questionnaire post lung cancer diagnosis. It is possible that the conditions were early manifestations or symptoms of lung cancer that were mis-diagnosed particularly for emphysema and chronic bronchitis. This is less probable for pneumonia or tuberculosis, however, tumors may have been interpreted as lesions from infections prior to cancer diagnoses.

As an inherited limitation of case-control studies, differential recall bias may account for part of the association observed. However, the issues of misclassification and recall bias can be adequately addressed in the cohort studies included in the analyses. Also, several of the cohort studies employed quantitative diagnostic tools such as a measure of forced expiratory volume to diagnose COPD. This may have reduced the potential for misclassification bias. Among these studies the effects were stronger than the results based on the self-reported medical history, suggesting that recall or misclassification bias do not explain the association with COPD and may in fact underestimate the true effect. A previous meta-analysis of exclusively FEV_1_ among prospective studies with over 5,000 participants calculated a combined estimate of 2.23 fold (95% CI: 1.93, 8.25) when comparing the highest quintile of FEV_1_ to the lowest among the 4 studies included [98].

Several additional sources of potential biases are noteworthy within the individual studies. The prevalence of previous lung diseases among controls was often much higher than the baseline rates reported in the general population, particularly in those studies conducted in China, in which 9% [84] and 14% [92] prevalence as opposed to a point prevalence of pulmonary tuberculosis of 573 (95% CI 472–631) per 100 000 (1% prevalence) in a population survey conducted by the China Tuberculosis Control Collaboration [99] and a lifetime prevalence of less than 1% in the cohort examined [94]. This suggests the potential for selection or recall biases in these studies. Several studies used both direct and next of kin interviews, potentially leading to differential recall bias as next of kin of the deceased may have excessively ruminated over questions leading to a higher likelihood of a positive response among cases. It should also be noted that COPD may be under-diagnosed in North America [100].

Reverse causality must also be considered in the cases of pneumonia and tuberculosis as infections may have been the result of a weakened immune system due to lung cancer. For these conditions, ascertainment bias must also be considered as individuals with tuberculosis or pneumonia may have been more likely diagnosed with lung cancer due to the use of additional chest x-rays in the diagnostic work-up often used for the infections. The possibility of reverse causality was addressed in several studies by examining the time of infection prior to cancer diagnosis. Significant increases in risk were consistent in latency analyses even at greater than 10–20 years since diagnosis of TB [74], [92], [94]. Combining the latency evidence, although not perfectly consistent and comparable across studies, suggests that the diseases of interest are related to lung cancer risk after long exclusion/latency periods. Among those studies that included latency analyses [27], [28], [30], [47], [64], [65], [67], [68], [70], [74], [89], [92], we observed elevated estimates for >10 and >20 years prior to cancer diagnosis for chronic bronchitis, tuberculosis and chronic bronchitis, emphysema and COPD combined. For emphysema and pneumonia elevated estimates for >10 years were observed (results not shown), however not for >20 years. It is worth noting that only 20–30% of studies included, depending on the disease, conducted such analyses, therefore results should be cautiously interpreted. Although histology patterns of lung cancer have changed over time in the last few decades, we did not see any difference in the effects stratified by study period. (results not shown).

It is also possible that our results, particularly among never smokers, may have been due to confounding from another source such as second hand smoke (SHS). SHS has been associated with increased risk of lung cancer [101] and may be related to previous lung diseases [102]. The majority of the case-control studies examining the effects among never smokers have adjusted their analyses for SHS in an attempt to control for potential confounding, nevertheless, the possibility of residual confounding cannot be excluded. Also, occupational exposures may have acted as confounders in the associations tested as they have been associated with lung cancer [103], particularly among never smokers.

We found minimal evidence of publication bias for pneumonia using standard methodologies. Publication bias may have occurred in the examination of the previous respiratory conditions of interest as many studies only reported results for those conditions that showed a significant association. It appears that for COPD a surplus of large positive studies or a dearth of smaller negative studies lead to significant tests. Publication bias was suggested for tuberculosis. This may be attributable to several of the smaller Asian studies reporting very large effect estimates. Another possibility is that Asian studies where small or null effects were observed not being published in English journals and as such omitted from this data collection.

The previous lung diseases examined in this meta-analysis as a group affect a large population of individuals. In the United States, the conditions have a prevalence of: for emphysema 18.5 per 1000 people, for chronic bronchitis 43.0 per 1000 [9] and tuberculosis 4.8 per 100,00 [10] and although the actual incidence of pneumonia in the US is unknown there were an estimated 1.4 million hospital discharges from pneumonia infections in 2005 [104]. The positive associations between these conditions and lung cancer risk are of substantial public health importance due to the large population exposed.

In conclusion, we observed a consistent positive association between COPD, emphysema, chronic bronchitis, pneumonia and tuberculosis and lung cancer risk in this meta-analysis. The observation of consistent associations when effects were examined among studies of never smoking cases supports a direct association between the conditions and lung cancer, reducing the likelihood of confounding by tobacco exposure. The most likely explanation for the increased risk associated with these diseases is the inflammatory effects within lung tissue. Previous lung conditions are known to induce an inflammatory response in the lung [7]. Recent evidence has suggested that inflammation plays a pivotal role in the development of lung cancer [97], [105], [106], particularly among never smokers. Inflammation may increase the risk of cancer development as an initiator or promoter through three processes; increased genetic mutations, anti-apoptotic signaling [107] and increased angiogenesis [8]. Further investigations into the potentially causal mechanisms whereby these conditions, promote lung cancer development are warranted. As such, larger studies or pooled analyses with the ability for standardized adjustment and more detailed subgroup analyses would be better suited to address these issues.

## Supporting Information

Figure S1 **Galbraith radial plot of the effects of chronic obstructive pulmonary disease, chronic bronchitis and emphysema across studies.**
(DOC)

Figure S2 **Galbraith radial plot of the effects of chronic bronchitis across studies.**
(DOC)

Figure S3 **Galbraith radial plot of the effects of chronic obstructive pulmonary disease across studies.**
(DOC)

Figure S4 **Galbraith radial plot of the effects of emphysema across studies.**
(DOC)

Figure S5 **Galbraith radial plot of the effects of pneumonia across studies.**
(DOC)

Figure S6 **Galbraith radial plot of the effects of tuberculosis across studies.**
(DOC)

Figure S7 **Funnel plot of the effects of chronic obstructive pulmonary disease, chronic bronchitis and emphysema across studies.**
(DOC)

Figure S8 **Funnel plot of the effects of chronic bronchitis across studies.**
(DOC)

Figure S9 **Funnel plot of the effects of chronic obstructive pulmonary disease across studies.**
(DOC)

Figure S10 **Funnel plot of the effects of emphysema across studies.**
(DOC)

Figure S11 **Funnel plot of the effects of pneumonia across studies.**
(DOC)

Figure S12 **Funnel plot of the effects of tuberculosis across studies.**
(DOC)

Table S1 Study characteristics of all studies included in the meta-analysis.
(DOC)
