# Supplementary figures and images for: Previous Lung Diseases and Lung Cancer Risk: A Systematic Review and Meta-Analysis
Source: PLoS One. 2011 Mar 31;6(3):e17479. doi: 10.1371/journal.pone.0017479 (PMC3069026; doi:10.1371/journal.pone.0017479)

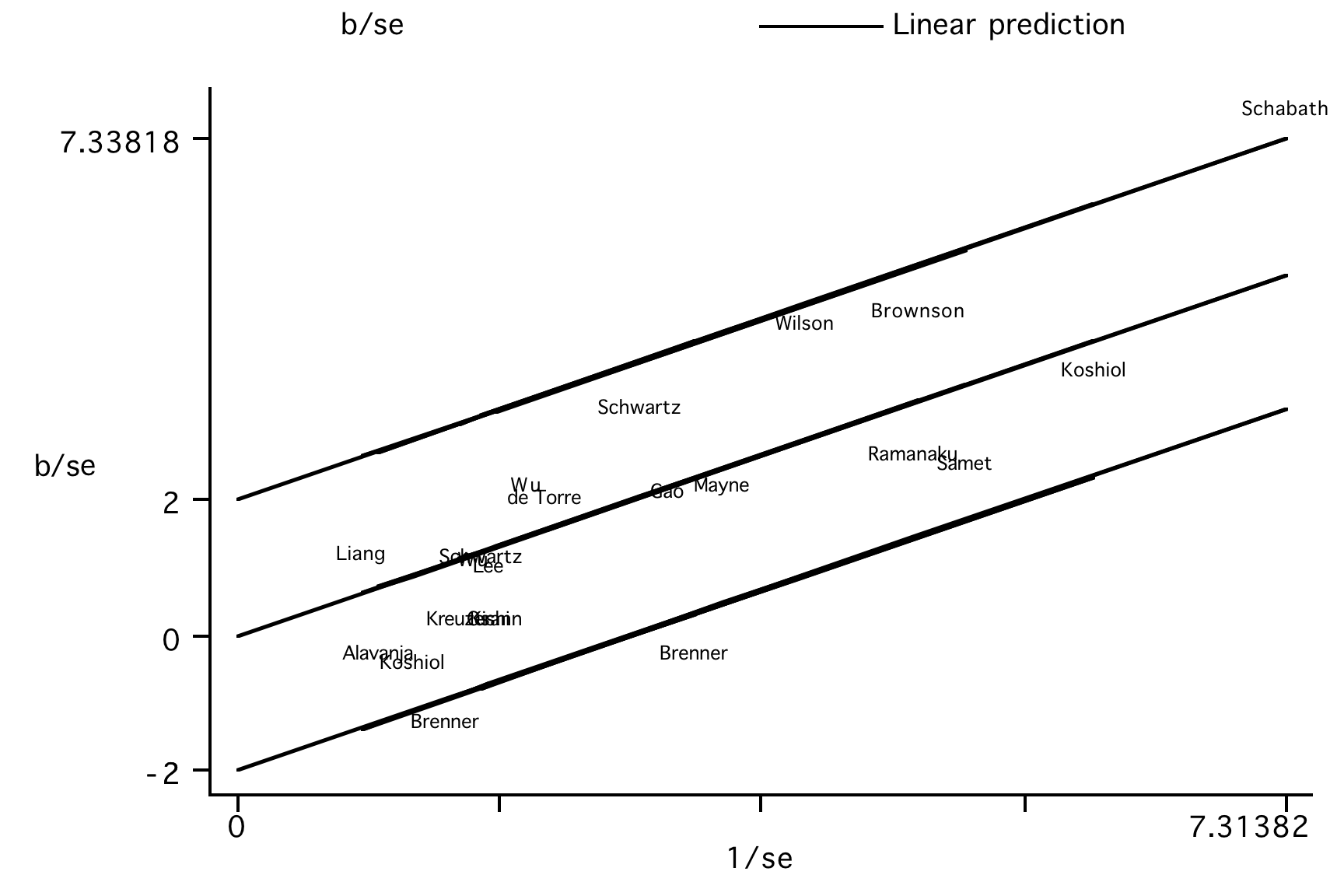


Supplementary Figure S4. Galbraith radial plot of the effects of emphysema across studies

Supplement: Figure S4 — Galbraith radial plot of the effects of emphysema across studies. (DOC) [file pone.0017479.s004.doc]

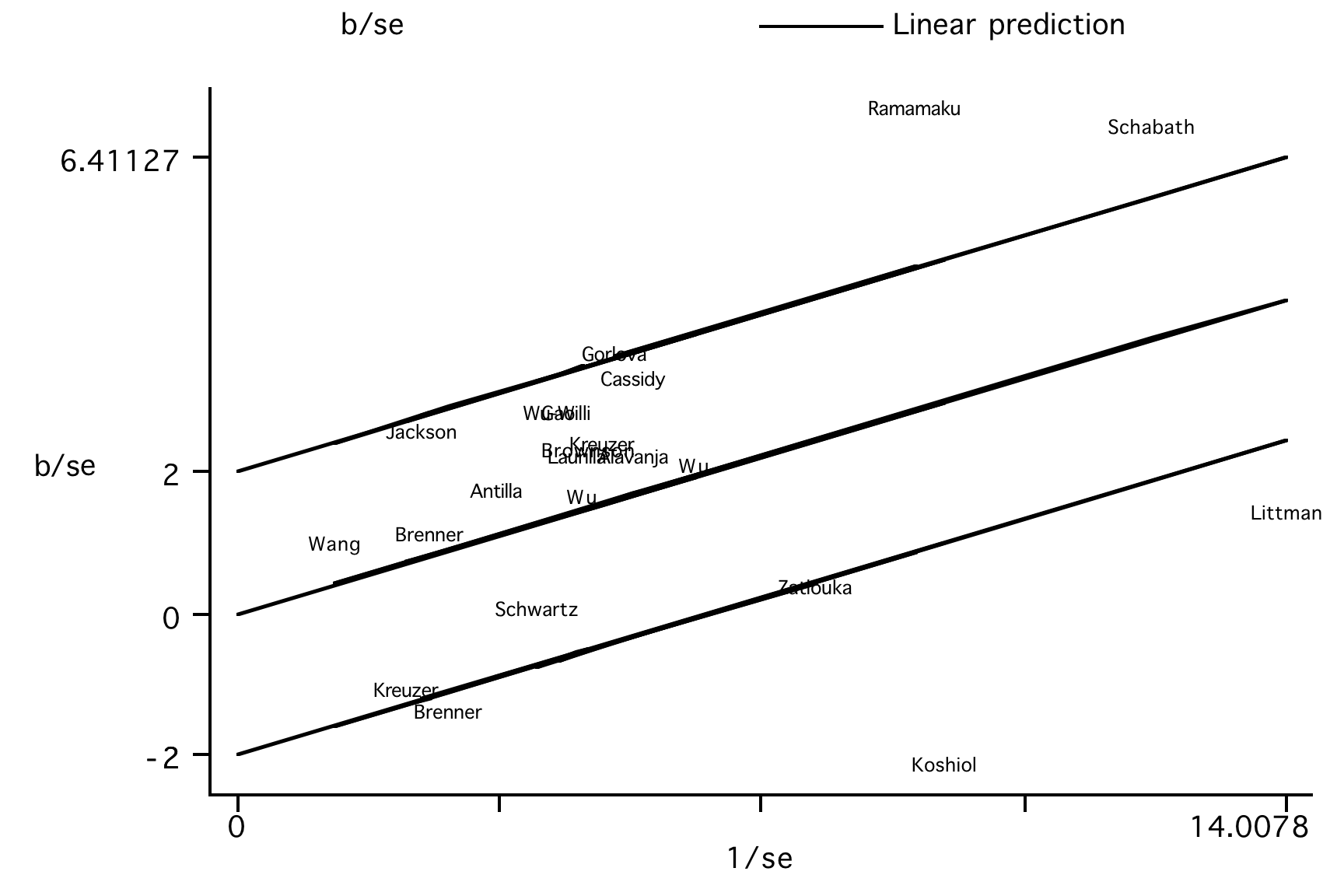


Supplementary Figure S5. Galbraith radial plot of the effects of pneumonia across studies

Supplement: Figure S5 — Galbraith radial plot of the effects of pneumonia across studies. (DOC) [file pone.0017479.s005.doc]

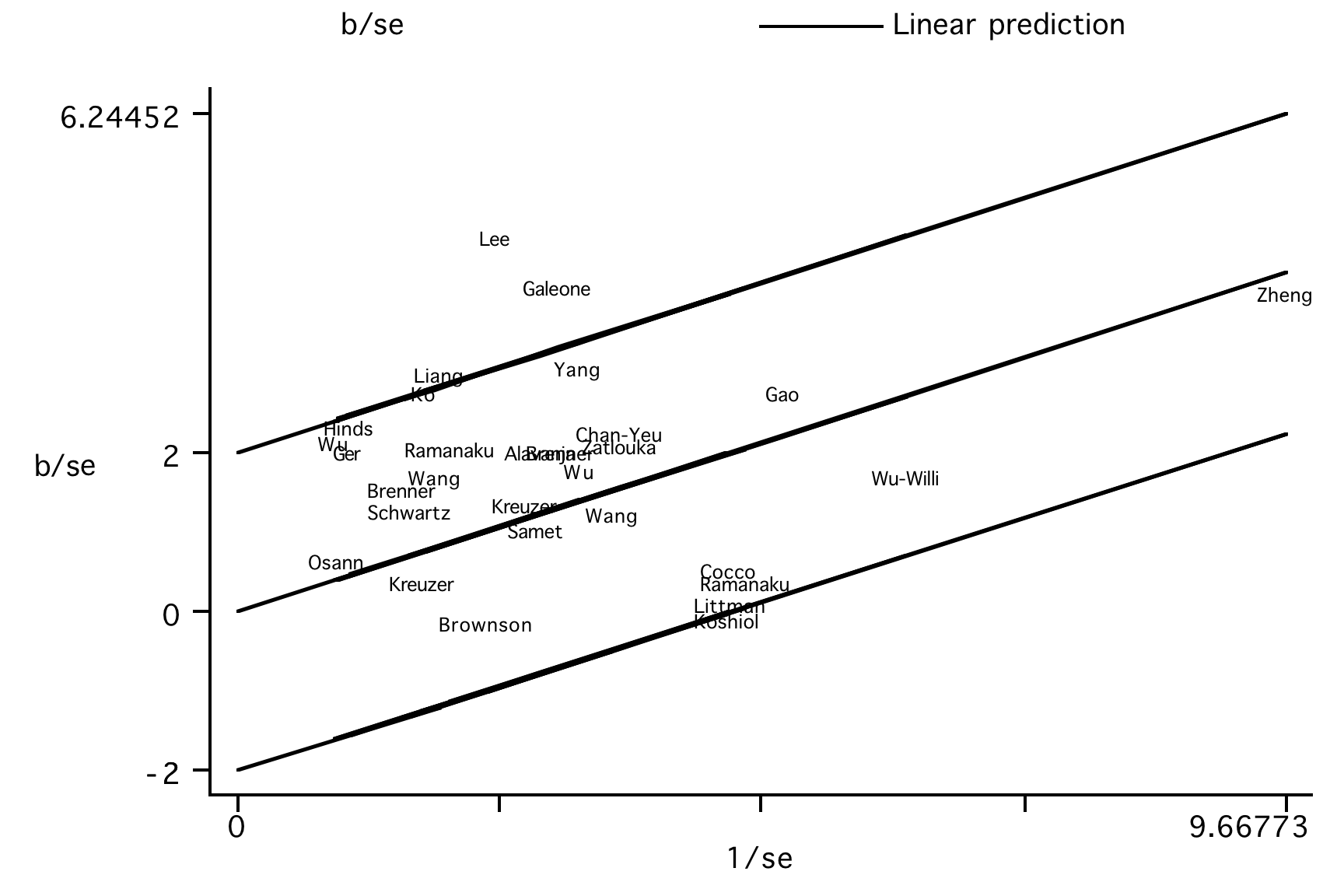


Supplementary Figure S6. Galbraith radial plot of the effects of tuberculosis across studies

Supplement: Figure S6 — Galbraith radial plot of the effects of tuberculosis across studies. (DOC) [file pone.0017479.s006.doc]
