# Supplementary material for: Previous Lung Diseases and Lung Cancer Risk: A Systematic Review and Meta-Analysis
Source: PLoS One. 2011 Mar 31;6(3):e17479. doi: 10.1371/journal.pone.0017479 (PMC3069026; doi:10.1371/journal.pone.0017479)
Supplement: Figure S7 — Funnel plot of the effects of chronic obstructive pulmonary disease, chronic bronchitis and emphysema across studies. (DOC) [file pone.0017479.s007.doc]

Supplementary Figure S7. Funnel plot of the effects of chronic obstructive pulmonary disease, chronic bronchitis and emphysema across studies
