# Supplementary material for: Previous Lung Diseases and Lung Cancer Risk: A Systematic Review and Meta-Analysis
Source: PLoS One. 2011 Mar 31;6(3):e17479. doi: 10.1371/journal.pone.0017479 (PMC3069026; doi:10.1371/journal.pone.0017479)
Supplement: Table S1 — Study characteristics of all studies included in the meta-analysis. (DOC) [file pone.0017479.s013.doc]

Table S1. Study characteristics of all studies included in the meta-analysis

| **Location and time period for diagnosis of lung cancer**  **(Author, Year)** | **PLDs examined**  **(diagnosis)** | **Number of cases** | **Case source/description** | **Cohort size / number of controls** | **Cohort/control source/description** | **Cohort/control**  **type** | **Matching factors** | **Adjustment**  **variables** | **Ref.** |
| --- | --- | --- | --- | --- | --- | --- | --- | --- | --- |
| **Cohort studies** |  |  |  |  |  |  |  |  |  |
| Sweden, 1971-2001  (Purdue, 2007) | COPD  (%FEV_1_) | 834 | Country cancer registries, Occupational cohort of Swedish construction workers | 176 997 | Occupational cohort of Swedish construction workers | Occupational | -- | Age, smoking, lung function | [47] |
| Denmark, 1974-1985  (Vestbo, 1991) | Chronic bronchitis  (self report) | 35 | Danish Cancer Registry | 876 | Random sample of men living in the city of Aalborg in 1973. | Population sample | -- | Age and tobacco consumption | [87] |
| USA, 1971-1992  (Mannino, 2003) |  | 113 | Hospitalization records, vital status, and death certificates of cohort | 5 402 | National Health and Nutrition Examination Survey | Population sample | -- | Age, sex, race, smoking status, pulmonary function level, pack-years of cigarettes, years since last smoking | [46] |
| (USA 1978-1990)  (Nomura, 1991) | COPD  (%FEV_1_) | 172 | SEER registry | 6317 | Honolulu Heart Program | Men of Japanese ancestry | -- | Age, age started smoking, if smoked number of cigarettes smoked per day | [49] |
| USA, 1985-2002  (Littman, 2004) | Chronic Bronchitis/  Emphysema  Tuberculosis  Pneumonia  *(self report)* | 1028 | Cancer registries, state boards of health and National Death Index | 17 698 | Carotene and Retinol Efficacy Trial (CARET) | High risk individuals | -- | Sex, Study arm, education, BMI, years smoked, years smoked, avg. number of cigarettes smoked,  avg. number of cigarettes smoked | [67] |
| Spain, 2000-2005  (de Torres, 2007) | Emphysema  (Low Dose CT) | 23 | Screening study for LDCT at the University of Navarra | 1166 | Non-cases in screening trial | >40 years of age, >10 pack-years smoking, no lung cancer symptoms | -- | Age, gender and nmber of pack-years of smoking | [50] |
| China, 1976-1996  (Engels, 2009) | Tuberculosis | 2 459 | Death records from hospitals, public security bureaus and public health records | 42 422 | Administrative records of famers born during 1917-1951 living in Xuanwei | Population based | -- | Lung disease and smoking | [94] |
| USA, 2002-  (Wilson, 2008) | COPD  Emphysema | 99 | Participants in the Pittsburgh Lung Screening Study | 3642 | Non-cases in screening trial | 50-79 years of age, current or ex smokers, no personal history of cancer, <400 lbs, no CT scans within 12 months | -- | Age, sex, years of smoking and smoking dose intensity | [51] |
| USA, 2002-2007  (Mortensen, 2010) | COPD | 3760 | A cohort of patients that have been hospitalized previously for pneumonia | 40 744 | Those patients that never developed lung cancer | 65 years or older with a previous diagnosis in the clinic for pneumonia in the past year |  | age at admission, race/ethnic group, tobacco use and marital status | [71] |
| **Case-control studies** |  |  |  |  |  |  |  |  |  |
| Finland, 1985-1993  (Laurila, 1997) | Pneumonia  *(Serological C. pneumoniae IgA≥16)* | 426 | Cohort of 29,133 randomized into prevention trial, Finnish cancer registry | 4226 | No disease within cohort | High risk cohort – population | Age, study centre, treatment group, timing of samples | Daily number of cigarettes, years of smoking | [52] |
| USA, 1987-2000  (Littman, 2004) | Pneumonia  *(Serological C. pneumoniae IgA≥16)* | 508 | Cohort of 18,314 randomized into prevention trial,  Cancer Registries, National Death Index | 508 | No cancer under observation in trial |  | Year of randomization, age at random., study arm, exposure cohort, smoking status | Education, BMI, years smoked, cigarettes smoked per day | [55] |
| USA, 1999  (Jackson, 2000) | Pneumonia  *(Serological C. pneumoniae IgA≥16)* | 143 | Larger case-control designed to examine the risk of lung cancer among workers in the wood industry | 147 | Random digit dialing in target area | Population based | Gender, 5-year age groups | Age, smoking status, pack-years, educations | [53] |
| Finland, 1983-2000  (Anttila, 2003) | Pneumonia  *(Serological C. pneumoniae IgA≥16)* | 58 | Cohort of 650,000  Finish cancer registry | 287 | Women in the same cohort free of cancer | Population based | Individually matched (1:5) for sex, age (±2 years) and sample storage time | Current smoking (serum cotinine) | [54] |
| China, 1984-1986  (Gao, 1987) | Chronic Bronchitis  Tuberculosis  Pneumonia  Emphysema  *(self report)* | 672 | Shanghai Cancer Registry | 735 | Random sample of Shanghai population | Population based | Sex, 5-year age groups | Smoking categories, age and education | [73] |
| USA, 1968-1978  (Hinds, 1982) | Tuberculosis  (x-ray) | 211 | Hawaii tumor registry | 419 | Population | Population | Year of birth (±3 years), race, sex, smoking history and birthplace | - | [56] |
| China, 1984-1986  (Zheng, 1987) | Tuberculosis  *(Self-report)* | 1,405 | Shanghai Cancer Registry | 1,495 | Random sample of Shanghai population | Population | 5-year age groups | Smoking categories, age and education | [92] |
| USA, 1980-1982  (Samet, 1986) | Chronic Bronchitis  Tuberculosis  Pneumonia  Emphysema  *(self report)* | 521 | Incident cases from New Mexico Tumor Registry | 769 | Randomly generated residential telephone numbers. Older than 65yr, New Mexico Medicare files | Population | Frequency matched on age, sex and ethnicity | Age, sex, ethnicity and cigarette smoking | [62] |
| USA, 1983-1986  (Wu, 1988) | Bronchitis  Tuberculosis  Pneumonia  Emphysema  *(self report)* | 336 | Los Angeles County Cancer Surveillance Program | 336 | From neighborhood of case | Population | Individually matched on race and date of birth (±5 years) | Pack-years, years since stopped smoking, depth of inhalation | [63] |
| USA, 1982-1984  (Mayne, 1999) | Chronic Bronchitis  Tuberculosis  Emphysema  *(self report)* | 437 | Incident cases in 23 counties of upstate New York – rapid case ascertainment system | 437 | New York State Department of Motor Vehicles’ file of licensed drivers | Population | Individually matched on age (±5 years), age, sex and county of residence | Cigarettes smoked/day, lifetime passive smoke exposure | [66] |
| USA, 1985-1988  (Wu, 1995)  (Never smokers) | Chronic Bronchitis  Tuberculosis  Pneumonia  Emphysema  *(self report)* | 412 | Incident case in Atlanta, Houston, New Orleans, San Francisco and Los Angeles County | 1253 | Population controls from random digit dialing | Population | Frequency matched on age group, ethnicity, study area, and lifetime nonusers of tobacco | Age groups, ethnicity and education | [64] |
| USA, 1986-1991  (Alavanja, 1992) | Chronic Bronchitis  Tuberculosis  Pneumonia  Emphysema  *(self report)* | 618 | Incident cases in the Missouri Cancer Registry | 1405 | Population-based sample of white, non-smoking female, from driver’s license files | Population | Frequency matched on 5-year age strata | Age, smoking history | [17] |
| China, 1994-1998  (Brenner, 2001) | Chronic Bronchitis/  Emphysema  Tuberculosis  Pneumonia  *(self report)* | 886 | Incident cases diagnosed in local hospitals in the Gansu province | 1765 | Randomly sampled from population census list for two prefectures | Population | Frequency matched by gender, 5-year age group and prefecture | Age, gender, prefecture, active smoking | [74] |
| China, 1985-1987  (Wu-Williams, 1990) | Chronic Bronchitis/  Emphysema  Tuberculosis  Pneumonia  *(self report)* | 965 | Cancer Registries of Harbin and Shenyang | 959 | Random selection from local population | Population | Frequency matched by 5-year age group | Age, education, smoking, study area | [84] |
| USA, 1995-2003  (Schabath, 2005) | Bronchitis  Emphysema  Pneumonia  *(self report)* | 1553 | Incident cases from the University of Texas M.D. Anderson Cancer Centre. | 1375 | Healthy controls without previous diagnosis of cancer from physician clinics | Hospital | Frequency matched to the cases on age (±5 years), gender, ethnicity and smoking status | Age, gender, ethnicity, smoking status, pack-years, occupational exposure to asbestos and wood dust | [68] |
| Czech Republic, 1998-2002  (Zatloukal, 2003) | Tuberculosis  Pneumonia  *(self report)* | 366 | Incident cases in hospitals of North-eastern Prague | 1624 | Healthy women visiting husbands in hospital | Hospital | - | Age, education, residence, pack-years of smoking | [90] |
| Taiwan, 1992-1993  (Ko, 1997) | Chronic bronchitis  Tuberculosis  *(Self report)* | 117 | Kaohsiung Medical College Hospital | 117 | Women visiting ophthalmic department in hospital | Hospital | Age (±2 years) | SES, residential area and education | [77] |
| Taiwan, 1993-1999  (Lee, 2001)  (Never smokers) | Chronic bronchitis  Tuberculosis  *(Self report)* | 527 | Kaohsiung Medical College Hospital | 805 | Hospitalized patients at same hospital with unrelated conditions | Hospital | Frequency matched on sex, age (±2 years) | Smoking, residential area and SES | [78] |
| USA, 1990-1993  Osann, 2000) | Chronic Bronchitis/  Emphysema  Tuberculosis  Pneumonia  *(self report)* | 98 | Orange County Hospitals | 204 | Random-digit dialing in Orange county and Long Beach | Population | Frequency matched for age | Age, education and smoking | [39] |
| Canada, 1979-1986  (Ramanakumar, 2006) | Emphysema  Tuberculosis  Pneumonia  *(self report)* | 775 | Metropolitan hospital in Montreal | Pop’n = 512  Cancer =1204 | Randomly sampled from population based electoral lists | Population & Hospital (cancer controls) | Frequency matched for sex, age | Age, ethnicity, type of respondent, years of school, income, smoking | [70] |
| Canada, 1995-2001  (Ramanakumar, 2006) | Emphysema  Tuberculosis  Pneumonia  *(self report)* | 1205 | Metropolitan hospital in Montreal | 1541 | Randomly sampled from population based electoral lists | Population | Frequency matched for sex, age | Age, ethnicity, type of respondent, years of school, income, smoking | [70] |
| Taiwan, 1990-1991  (Ger, 1993) | Chronic bronchitis  Tuberculosis  *(Self report)* | 131 | Tri-Service General Hospital, in Taipei | 524 | 262 Hospital controls 262 randomly selected neighborhood controls | Hospital | Frequency matched sex, date of birth (±5 years), date of interview, insurance status | Cigarette smoking, asbestos related job, use of coal, passive smoking | [76] |
| China, 1972-1989  (Cocco, 200) | Chronic bronchitis  Tuberculosis  *(Self report)* | 316 | Occupation cohort in China | 1356 | In the cohort alive at the time of index case | Nested – Population | Individually matched by decade of birth and facility type | Cigarettes per day | [93] |
| USA, 1999  (Kishi, 2002) | COPD  (%FEV_1_)  Emphysema  (quant. CT) | 24 | CT screening trial at Mayo Clinic Rochester | 96 | In the screening trial at end of follow-up without cancer | Hospital | Frequency matched sex, age and pack-years of smoking | Current-smoking status and duration of abstinence since quitting | [45] |
| USA, 1993-1994  (Brownson, 2000) | Chronic Bronchitis  Tuberculosis  Pneumonia  Emphysema  *(self report)* | 676 | Incident cases in the Missouri Cancer Registry | 700 | Population-based sample of white, non-smoking female, from driver’s license files. Among aged 65-84 years, from Health Care Financer Administration’s roster of Medicare recipients | Hospital | Frequency matched on 5-year age strata | Pack-years of smoking | [65] |
| Germany, 1991-1996  (Kreuzer, 2002)  (Never smokers) | Chronic Bronchitis/  Emphysema  Tuberculosis  Pneumonia  *(self report)* | 234 | Incident cases that were residents of defined areas in East and West Germany | 535 | Randomly selected from the general population from mandatory registries or by random-digit dialing | Hospital | Frequency matched on sex, age and region | Age, region | [85] |
| Germany, 1990-1996  (Kreuzer, 2001)  (Never smokers) | Chronic Bronchitis/  Emphysema  Tuberculosis  Pneumonia  *(self report)* | 58 | Incident cases that were residents of defined areas in East and West Germany | 803 | Randomly selected from the general population from mandatory registries or by random-digit dialing | Hospital | Frequency matched on sex, age and region | Age, region | [86] |
| China, 1990-1993  (Wang, 1996) | Chronic Bronchitis/  Emphysema  Tuberculosis  *(self report)* | 390 | Current inpatients from five hospital of the Universities of the city of Guangzhou | 390 | Non-malignant patients who were hospitalized during the same time period in the same hospital as the cases | Hospital | Frequency matched on sex, place of residence, education and age (± 5 years) | Family history, smoking, SHS exposure, consumption of pickled and cured foods | [75] |
| USA, 1995-2003  Gorlova, 2006  (Never smokers) | Pneumonia  *(self report)* | 280 | Histologically confirmed cases recruited at MD Anderson Cancer Centre | 242 | Private multi-specialty clinics in the greater Houston area | Hospital | Frequency matched for sex, ethnicity and age (± 5 years), | Age, gender, ethnicity, income and years of education | [89] |
| USA, 1984-1987  (Schwartz, 1996)  (Never smokers) | COPD  Emphysema  Chronic Bronchitis  Pneumonia  Tuberculosis  (*self report)* | 257 | Incident nonsmoking cases, 40-84 years of age from Occupational cancer study | 277 | Selected from population-based controls collected for large study, randomly selected | Population | Frequency matched on sex, ethnicity and age (± 5 years), | Age, gender, ethnicity, income education, family history, occupation in unusual industry | [69] |
| China, 1985-1986  (Liu, 1991) | Chronic Bronchitis | 110 | Incident cases diagnosed at Xuanwei hospitals and clinic | 426 | Selected within same hospital and clinics | Hospital | Individually matched based on age (± 5 years), sex and village of residence | Matching factors, smoking and cooking methods | [81] |
| Hong Kong, 1999-2001  (Chan-Yeung, 2003) | Tuberculosis  (self report) | 341 | Incident histologically confirmed cases from the Queen Mary Hospital | 341 | Selected from same hospital as cases – outpatient clinics | Hospital | Individually matched for age (± 5 years) and sex | Place of birth, education, family history and smoking and matching factors | [95] |
| Hong Kong, 2002-2004  (Wang, 2009)  (Never smokers) | Chronic Bronchitis  Pneumonia  Tuberculosis  (*self report)* | 212 | Incident histologically confirmed case among females from the largest oncology centre in Hong Kong | 292 | Selected from the same hospital with no history of physician-diagnosed cancer at any site | Hospital | Frequency matched on age (±10 years) | Age, employment, intakes of yellow/orange vegetables & dark green vegetables and multivitamins | [79] |
| China, 1987-1990  (Galeone, 2008) | COPD  Tuberculosis  (self report) | 218 | Incident histologically confirmed cases from Cardiothoracic surgery dept | 436 | Patients admitted to the general, cardiothoracic and urological, orthopedic surgery depts. For non noeplastic non lung diseases | Hospital | Individually matched on age (± 5 years) and area of residence | Sex, age groups, area of residence smoking status, duration and amount of smoking, income, family history of lung and other cancers and occupational exposures | [80] |
| China, 1992-1994  (Wang, 1996)  (Never smokers) | Tuberculosis | 135 | Incident histologically confirmed cases in 18 hospitals in the city of Shenyang lifetime nonsmoker females | 135 | Females randomly selected from the general population located in urban areas of Shenyang | Population | Individually matched on age (± 5 years) | - | [96] |
| Morocco 1996-1998  (Sasco, 2002) | Chronic Bronchitis  (self report) | 118 | Incident cases from the IBN Rochd Hospital | 235 | Selected from other patients admitted to hospital for conditions unrelated to lung cancer | Hospital | Individually matched on age, sex and place of residence | Matching factors and smoking status | [88] |
| China, 1986-1993  (Shen, 1996) | Chronic Bronchitis  (self report) | 80 | Primary lung cancer cases from Nanjing Municipal Hospitals | 180 | Residents of Nanjing 20 years old and tumor-free | Population | Frequency matched on sex, age (± 5 years), nationality and street address | Matching factors, smoking index, cooking fumes, family tumor history | [82] |
| USA, 2001-2005  (Schwartz, 2009) | Chronic Bronchitis  Emphysema  COPD  Pneumonia | 562 | Primary non-small cell cancer through the Metro Detroit Cancer Surveillance | 564 | Identified through random digit dialing, | Population | Frequency matched on race and five-year age group. | Age, race, years of education, pack-years of smoking, current BMI, family history of lung cancer and regular use of aspirin | [30] |
| USA,  (Yang, 2008) | COPD | 1585 | Incident cases of lung cancer from the Mayo Clinic | 1585 | Community residents identified by having had a general medical exam and a leftover blood sample | Population | Individually matched on age, sex, and race/ethnicity | Matching factors and smoking status | [72] |
| China, 2004-2007  (Liang, 2009) | Tuberculosis  Chronic Bronchitis  Emphysema | 226 | Incident cases of lung cancer were identified in 18 hospitals in Shenyang | 279 | Randomly selected from the general population of urban Shenyang | Population | Age (± 5 years) | Demographic characteristics, exposure to SHS, coal combustion and fumes | [83] |
| Canada, 1997-2002  (Brenner, 2010) | Emphysema  Chronic Bronchitis  Pneumonia Tuberculosis | 445 | Incident case from tertiary care hospitals in the Greater Toronto Area | 948 | Population – randomly selected from property tax assessment files  Hospital – Family medicine clinic | Mixed | Frequency matched based on age and sex | Pack-years of smoking, age, sex, education and ethnicity | [29] |
| Italy,  Koshiol, 2009 | Chronic Bronchitis  Emphysema  COPD | 2100 | Incident cases enrolled form 13 hospitals in Lombardy region | 2120 | Healthy control were randomly sampled from the Regional Health Service database | Population | Frequency matched based on age, sex and area of residence | Age, sex, region , pack-years, amount of cigarette smoking, other lung diseases | [28] |
| Italy,  Koshiol, 2009 | Pneumonia | 2100 | Incident cases enrolled form 13 hospitals in Lombardy region | 2120 | Healthy control were randomly sampled from the Regional Health Service database | Population | Frequency matched based on age, sex and area of residence | Age, sex, region , pack-years, amount of cigarette smoking, other lung diseases | [28] |
| UK,  Cassidy, 2008 | Pneumonia | 579 | Incident cases of histologically confirmed lung cancer between the age of 20-80 | 1157 | Selected from registers of general practioners in Liverpool | Population | Individually matched by year of birth (± 5 years) and gender | Age, sex, smoking duration, asbestos exp., prior history of cancer, family history | [91] |
|  |  |  |  |  |  |  |  |  |  |

*BMI* body mass index, *SHS* second hand smoke, *Pop’n* population, *COPD* chronic obstructive pulmonary disease, *%FEV_1_* forced expiratory volume in one second as a percentage of forced expiratory capacity, *quant CT* quantitative computer tomorography, *SES* socioeconomic status, *CT* computer tomography
